# A new protocol for long‐term culture of a specific subpopulation of liver cancer stem cells enriched by cell surface markers

**DOI:** 10.1002/2211-5463.12932

**Published:** 2020-07-31

**Authors:** Biao Zhang, Hai‐Yang Wang, Dong‐Xing Wang, Quan Zeng, Zeng Fan, Jia‐Fei Xi, Xue Nan, Li‐Juan He, Jun‐Nian Zhou, Xue‐Tao Pei, Wen Yue

**Affiliations:** ^1^ Stem Cell and Regenerative Medicine Lab Institute of Health Service and Transfusion Medicine Beijing China; ^2^ South China Research Center for Stem Cell & Regenerative Medicine SCIB Guangzhou China; ^3^ Experimental Hematology and Biochemistry Lab Beijing Institute of Radiation Medicine Beijing China

**Keywords:** cell surface markers, chemically defined medium, hepatocellular carcinoma, *in vitro* culture model, liver cancer stem cells

## Abstract

Liver cancer stem cells (L‐CSCs) are considered to be an important therapeutic target for hepatocellular carcinoma (HCC). This study provides a new *in vitro* long‐term culture model for a specific subpopulation of L‐CSCs enriched by cell surface markers. We combined CD13, CD133 and EpCAM to selectively enrich L‐CSCs, which we then cultured in modified chemically defined medium. The enriched L‐CSCs exhibited enhanced proliferation, self‐renewal and long‐term clonal maintenance ability as compared with non‐CSCs. Compared with wild‐type hepatocellular carcinoma, the expression of stemness surface markers, oncogenes, drug resistance and tumorigenicity in enriched L‐CSCs was significantly increased. In summary, the subpopulation of L‐CSCs still maintains cancer stem cell‐related phenotypes after 14 days of culture.

## Graphical abstract

We enriched a specific subpopulation of liver cancer stem cells using multiple surface markers and then cultured the cells with modified chemically defined medium to maintain the cancer stem cell properties for 2 weeks *in vitro*. This study may provide an effective *in vitro* model for the study of the biological properties of liver cancer stem cells and their use in targeted drug screening.

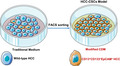

**Abbreviations**
bFGF: basic fibroblast growth factor
CDM: chemically defined medium
CSC: cancer stem cell
DMEM: Dulbecco's modified Eagle's medium
EGF: epidermal growth factor
elda: extreme limiting dilution analysis
FACS: fluorescence‐activated cell sorting
HCC: hepatocellular carcinoma
L‐CSC: liver cancer stem cell
MFI: mean fluorescence intensity
NOD/SCID: nonobese diabetic/severe combined immunodeficiency

Primary liver cancer in human is the fifth most common cancer and is the second leading cause of cancer‐related death worldwide. More than 90% of primary liver cancers are hepatocellular carcinoma (HCC) [1]; although traditional chemotherapy can significantly reduce the tumor bulk and mass, patients with liver cancer often die of recurrence and distant metastasis. Liver cancer stem cells (L‐CSCs) are thought to play an important role in drug resistance, tumor relapse and metastases of HCC [2]. L‐CSCs are a small subpopulation of stem‐like cancer cells; with a high self‐renewal and multidifferentiation abilities, they can drive and sustain tumor growth and reconstitute tumors in distal organs [3]. Targeting L‐CSCs has become an important topic in the treatment of HCC, and the establishment of the L‐CSC model *in vitro* is urgently needed [4]. To identify L‐CSCs, many surface markers are developed to isolate L‐CSCs by fluorescence‐activated cell sorting (FACS) or antibody‐conjugated magnetic beads, such as CD133, CD13, EpCAM, CD44, CD90, among others. However, in recent years, there are growing concerns that a single marker cannot separate the L‐CSCs in different HCC cell lines [5]. So far, there is not a ‘one‐fits‐all’ marker for L‐CSCs in most HCC cell lines. This suggests that combinations of multiple markers may be needed to identify L‐CSCs from different genetic subclones [6]. Moreover, L‐CSCs isolated by cell surface markers will gradually differentiate into the bulk of cancer cells in traditional serum‐contained medium in several days [7, 8, 9]. Therefore, it is difficult to study L‐CSCs in this way for a long time. In addition to cancer stem cell (CSC) surface markers, tumor sphere culture is another widely recognized method for the separation and enrichment of CSCs [10]. It requires both ultra‐low‐attachment culture surface and serum‐free media supplemented with growth factors, such as epidermal growth factor (EGF) and basic fibroblast growth factor (bFGF). Actually, tumor spheres contain mixed subpopulations of CSCs [11, 12]; even single‐cell‐derived tumor spheroid cultures are heterogeneous for CSC marker expression after differentiation induction [13], and these heterogeneous subpopulations are different in stemness and related functions. It is difficult to study a specific subpopulation of L‐CSCs and its properties. In view of this, there is an urgent need for an *in vitro* tumor stem cell model that can maintain the properties of a specific subpopulation of L‐CSCs.

There is growing evidence that supports the origination of L‐CSCs from malignant transformation of liver stem or progenitor cells [14]. There are many similar characteristics between L‐CSCs and normal hepatic progenitor cells, such as stemness‐related pathways, self‐renewal and multidifferentiation abilities [15]. In the study of normal liver stem cells, hepatic progenitor cells are cultured in modified chemically defined medium (CDM), which is a kind of serum‐free culture medium to expand and sustain stemness of hepatic progenitor cells [16]. In this case, we hypothesized that the modified CDM is likely to play a similar role in the expansion and stemness maintenance of L‐CSCs. This may be of benefit for maintaining the properties of a specific subpopulation of L‐CSCs, which is of great significance for the study of L‐CSCs.

In this study, three kinds of L‐CSC surface markers were combined to selectively enrich L‐CSCs in HCC cell lines by FACS. These enriched L‐CSCs were cultured in the modified CDM. We evaluated the effects of this *in vitro* model on maintaining stemness properties of the specific subpopulations of L‐CSCs by detecting the changes of CSC‐related markers and functions. This L‐CSC‐cultured system will provide new ideas and methods for the study of L‐CSCs and the screening of targeted drugs.

## Materials and methods

### Cell culture

The Huh7.5.1 cells were maintained in our laboratory. Hep3B and PLC/PRF/5 cells were obtained from American Type Culture Collection (Manassas, VA, USA). Mycoplasma testing has been done for the cell lines used. Huh7.5.1 and PLC/PRF/5 cells were cultured in Dulbecco's modified Eagle's medium (DMEM) with 10% FBS, and Hep3B cells were cultured in minimum Eagle’s medium with 10% FBS. The modified CDM for enriched L‐CSCs consists of 1 : 1 DMEM/F12 and Neurobasal medium, 100 μg·mL^−1^ penicillin‐streptomycin, 2 mmol l‐glutamine, 0.1 mm 2‐mercaptoethanol, 1 : 200 N2 (GIBCO, Grand Island, NY, USA), 1 : 100 B27 (GIBCO), 0.1% BSA, 10 ng·mL^−1^ transforming growth factor‐α, 10 ng·mL^−1^ BMP4, 15 ng·mL^−1^ bFGF (PeproTech, Rocky Hill, NJ, USA), 15 ng·mL^−1^ EGF (R&D Systems, Minneapolis, MN, USA) and 20 ng·mL^−1^ hepatocyte growth factor. All cells were cultured at 37 °C and 5% CO_2_. The relevant reagents were listed in Table S1.

### Flow cytometry analysis and sorting

HCC cells were collected and resuspended with PBS. The antibodies (anti‐human CD13‐Peridinin‐Chlorophyll‐Protein Complex‐CyTM5.5 Ig, anti‐human EpCAM‐Allophycocyanin Ig, anti‐human CD133‐Phycoerythrin Ig and their corresponding anti‐human IgG) were diluted according to the manufacturer's instructions, then added to those HCC cells and incubated at 4 °C for 30 min. After antibody incubation, we washed the labeled HCC cells with PBS for three times, and the labeled HCC cells were analyzed and sorted by flow cytometry (BD Biosciences, San Jose, CA, USA). The relevant antibodies were listed in Table S2.

### Long‐term clonal growth assay

The CD13^+^CD133^+^EpCAM^+^ and CD13^−^CD133^−^EpCAM^−^ Hep3B cells were sorted to low‐adhesion 96‐well plates (single cell per well) at three gradients (32, 45 and 60 cells per plate). The single cell was cultured with modified CDM (100 μL per well) and added to 50 μL modified CDM every 4 days. After 4 weeks, the images of the clonal subpopulation formed in each group were captured and analyzed by extreme limiting dilution analysis (elda) software.

### Tumor sphere formation assay

HCC cells were planted on low‐adhesion six‐well plates (3000 cells per well) in serum‐free DMEM/F‐12 medium, which contained 1 : 100 N2, 1 : 50 B27, 10 ng·mL^−1^ EGF, 10 ng·mL^−1^ bFGF and 100 μg·mL^−1^ penicillin‐streptomycin. Two weeks later, the images of tumor spheres were captured and analyzed by image‐pro plus 6.0 (Media Cybernetics, Silver Springs, MD, USA). Three independent experiments were analyzed at least. The relevant reagents were listed in Table S1.

### Cell chemoresistance assay

A total of 3 × 10^3^ enriched L‐CSCs or wild‐type HCC cells per well were seeded in 96‐well plates with CDM media. After all cell attachment, different concentrations of chemotherapeutic drugs (doxorubicin: 0.4/0.8 μg·mL^−1^; sorafenib: 4/8 μm) were added to 96‐well plates for 48 h. Then we replaced medium with Cell Counting Kit‐8 (Dojindo Laboratories, Kumamoto, Japan) and incubated at 37 °C for 2–3 h. Absorbance at 450 nm was measured by SpectraMax M2e (Molecular devices, San Jose, CA, USA). Five replicate wells for each group and three independent experiments were conducted. The relevant kits were listed in Table S1.

### Immunofluorescence

In brief, the fixed cells were blocked with 10% BSA and incubated with diluted primary antibodies overnight at 4 °C according to the manufacturer's instructions, followed by incubation with diluted fluorescence‐conjugated secondary antibodies for 30 min (1 : 50 ZF‐3011 and 1 : 50 ZF‐3013; Zhongshan Bio‐Tech, Beijing, China). Then we stained nuclei with DAPI (hydrochloride) for 5 min and washed with PBS for three times. Fluorescent images were collected by ECLIPSE TE2000‐U microscope (Nikon Corporation Precision Equipment Company, Tokyo, Japan). The relevant antibodies were listed in Table S2.

### Animal studies

Nonobese diabetic/severe combined immunodeficiency (NOD/SCID) mice (male, aged 3–4 weeks) were purchased from Vital River Laboratories (Beijing, China). The mice were housed and handled according to protocols approved by the Beijing Medical Experimental Animal Care Commission. For tumorigenicity assay, 1 × 10^4^ wild‐type Hep3B parental cells and CDM‐cultured Hep3B‐CSC cells were injected subcutaneously into the left or right flanks of NOD/SCID mice, respectively. After 4 weeks, the mice were sacrificed, and the formed tumors were examined.

### Statistical analysis

The intensity of immunofluorescence staining and tumorsphere areas were analyzed by image‐pro plus 6.0 software. Statistical graphs were performed by graphpad prism 8.0 software (La Jolla, CA, USA). The mean of two random samples was analyzed by Student's *t*‐test, and the differences among multiple groups were statistically analyzed by two‐way ANOVA test. The results were presented as mean ± SEM. The following *P* values were regarded as statistically significant: **P < 0.01 or *P < 0.05.

## Results

### The enriched L‐CSCs after CDM culture presented stem cell‐like morphology and stronger proliferation than non‐CSCs

**Fig. 1:**
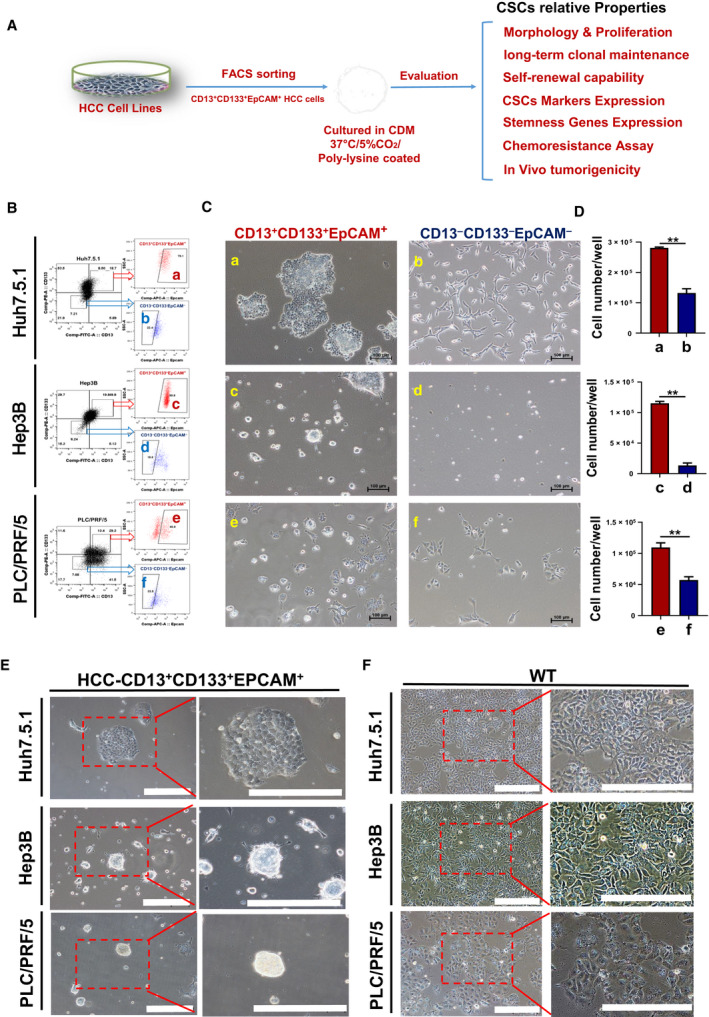
Morphology and proliferation of enriched L‐CSCs versus non‐CSCs in CDM. (A) Brief schematic diagram of L‐CSCs enriching, culturing and analysis in this study. (B) The HCC cells sorting by FACS: (a, c, e) CD13^+^CD133^+^EpCAM^+^ HCC cells and (b, d, f) CD13^−^CD133^−^EpCAM^−^ HCC cells. (C) Morphology of enriched L‐CSCs versus non‐CSCs cultured by modified CDM for 2 weeks. Scale bars: 100 μm. (D) Statistical histogram for proliferation of the two groups; *n* = 3, mean ± SEM. *P < 0.05; **P < 0.01, by Student's *t*‐test. (E, F) Morphology of enriched L‐CSCs cultured by modified CDM for 4 weeks versus that of wild‐type (WT) HCC cells. Scale bars: 200 μm.

To isolate L‐CSCs and non‐CSCs from HCC cell lines, we used three L‐CSCs surface markers, CD13, CD133 and EpCAM, to enrich triple‐positive and triple‐negative liver cancer cells from Huh7.5.1, Hep3B and PLC/PRF/5 cells by FACS. The enriched liver CSCs were cultured in the modified CDM for 2–4 weeks; then we detected and evaluated the characteristics of those enriched L‐CSCs (Fig. 1A).

First, we enriched CD13^+^CD133^+^EpCAM^+^ HCC cells by FACS as the L‐CSC group and cultured them in six‐well culture plates with modified CDM medium, and the surface of culture plates was coated with poly‐lysine, although the same number of CD13^−^CD133^−^EpCAM^−^ HCC cells was sorted as the non‐CSC group (Fig. 1B). After 2‐week culture, we observed the cell morphological changes and detected the proliferation of the two groups. The morphology of the L‐CSC group presented clonal aggregation growth, and these cells in clonal islands were small and densely arranged, which were in line with the morphological characteristics of stem cell clonal growth. However, the non‐CSC group was epithelioid and granular, with relatively diffuse distribution among the cells (Fig. 1C). Next, we compared the final cell number between the two groups (with equally initial cell number); the result showed that the proliferation ability of enriched L‐CSCs was significantly higher than that of non‐CSCs (Fig. 1D). After 4‐week culture, enriched L‐CSCs still maintain clonal island morphology, which is significantly different from the epithelioid morphology of wild‐type HCC (Fig. 1E,F).

### The enriched L‐CSCs have stronger self‐renewal and long‐term clonal growth ability than non‐CSCs during CDM culture

**Fig. 2:**
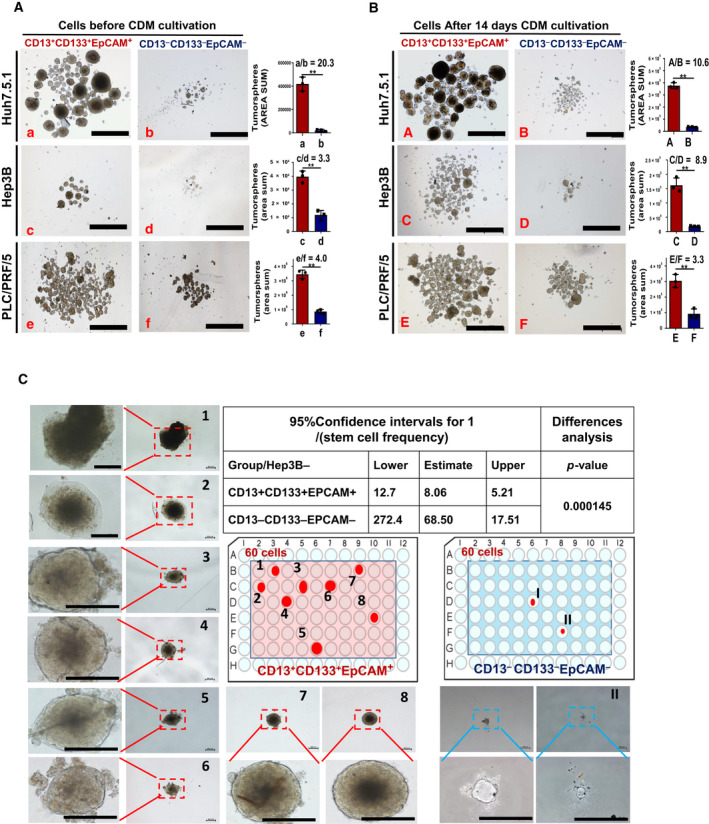
Sphere formation and long‐term clonal growth ability of enriched L‐CSCs. (A, B) Images and histogram of tumor sphere formation of enriched L‐CSCs versus non‐CSCs before (A) and after 14 days CDM culture (B). Scale bars: 1000 μm. *n* = 3, mean ± SEM. *P < 0.05; **P < 0.01, data analyzed by Student's *t*‐test. The sphere area sum mean ratio between L‐CSCs and non‐CSCs was listed in each histogram. (C) Long‐term clonal growth assay for single Hep3B‐CSC and Hep3B‐non‐CSC at 60‐cell gradient. Scale bars: 200 μm. The 95% confidence intervals of active L‐CSC frequency in two groups were analyzed by elda (http://bioinf.wehi.edu.au/software/elda/).

We performed sphere‐forming assay immediately without CDM culture on the same number of sorted L‐CSCs and non‐CSCs. The results showed that the self‐renewal ability of L‐CSCs was significantly higher than that of non‐CSCs (all *P* < 0.05), which is confirmed by the definition of CSCs (Fig. 2A). Then we cultured L‐CSCs and non‐CSCs in CDM for 14 days and carried out second‐round sphere‐forming assay. The results showed that the self‐renewal ability of L‐CSCs was still significantly higher than that of non‐CSCs (all *P* < 0.05). Compared with the assay before CDM culture, the number of non‐CSC‐derived tumor spheres increased slightly in all three HCC cell lines. The ratio of tumor spheres cross‐sectional area mean between L‐CSCs and non‐CSCs slightly decreased in Huh7.5.1 and PLC/PRF/5 cells, but significantly increased in Hep3B cells, because the number of Hep3B‐CSC‐derived spheres also significantly increased after CDM culture (Fig. 2B). Together, this culture system can stably maintain or promote the self‐renewal ability of L‐CSCs, even slightly promoting the self‐renewal ability of non‐CSCs.

One of the golden standard measurements of CSCs is maintenance of long‐term clonal growth in functional repopulation assays [17]. Here, we carried out single‐cell *in situ* tracking assay to compare the long‐term clonal growth ability of L‐CSCs and non‐CSCs. The single CD13^+^CD133^+^EpCAM^+^ Hep3B cell and CD13^−^CD133^−^EpCAM^−^ Hep3B cell were sorted into the 96‐well plate in CDM, respectively, in three sets of quantitative gradients (32, 45 and 60 cells per 96‐well plate) (Fig. 2C and Fig. S1A,B). After 4 weeks of culture, clonal islands were counted and results were analyzed by elda software, which is especially suitable for analyzing limiting dilution data [18]. The results showed that eight, five and four clonal islands were formed by Hep3B‐CSCs in three gradient groups (60, 45 and 32 cells per plate), respectively, whereas Hep3B‐non‐CSCs formed only two small clonal islands in the 60‐cell gradient (Fig. 2C and Fig. S1A,B). The elda analysis results showed that the frequency of Hep3B‐CSCs with long‐term clonal growth ability in L‐CSCs and the non‐CSC group were 1/8 and 1/68, respectively (*P* < 0.05) (Fig. 2C). The results indicated that the long‐term clonal growth ability of Hep3B‐CSCs cells was significantly higher than that of non‐CSCs during CDM culture.

### The enriched L‐CSCs can maintain the high expression of CSCs surface markers after CDM culture, except CD133 expression of PLC/PRF/5

**Fig. 3:**
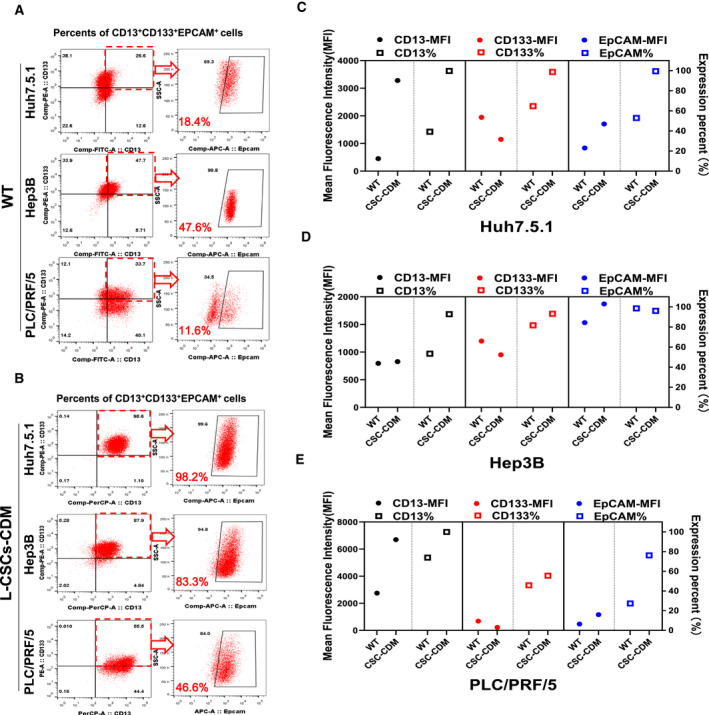
Surface markers expression of enriched liver CSCs cultured in modified CDM. (A, B) The CD13^+^CD133^+^EPCAM^+^ cell proportion of wild‐type HCC (A) and L‐CSCs cultured by CDM (B) were analyzed by FACS. (C–E) The scatter diagram of CD13, CD133 and EpCAM expression of wild‐type HCC cells (WT) and enriched L‐CSCs cultured in CDM (CSC‐CDM) for 14 days. The left *y* axis and solid dots represent MFI; the right *y* axis and hollow blocks represent expression proportion (%).

To investigate whether enriched L‐CSCs differentiated into the bulk of HCC cells after modified CDM culture, we detected the CD13^+^CD133^+^EpCAM^+^ cell proportion from enriched L‐CSCs cultured with CDM for 14 days and that of wild‐type HCC cells cultured with ordinary serum‐contained culture medium. The triple‐positive cells proportion from wild‐type Huh7.5.1, Hep3B and PLC/PRF/5 cells was 18.4%, 47.6% and 11.6%, respectively (Fig. 3A), while the triple‐positive cells proportion from enriched L‐CSCs in three HCC cell lines was 98.2%, 83.3% and 46.6%, respectively (Fig. 3B). Among these results, the enriched L‐CSCs could still maintain a high expression level of cell surface markers in Huh7.5.1 cells and Hep3B cells, except PLC/PRF/5 cells, which is mainly caused by the down‐regulation of CD133 expression. Nevertheless, it was still significantly higher than that of wild‐type PLC/PRF/5 cells (Fig. 3A,B).

To further clarify the expression changes of each L‐CSC surface marker between wild‐type HCC cells and enriched L‐CSCs cultured with CDM. We detected the changes of surface marker expression proportion and mean fluorescence intensity (MFI) of HCC cells before sorting and 2‐week CDM culture after sorting. The results showed that the CD13 expression proportion of sorted L‐CSCs from three HCC cell lines still maintained at a high level after the modified CDM culture, and the MFI of CD13 was significantly increased, except that of Hep3B, which was slightly up‐regulated (Fig. 3C–E). The expression proportion of EpCAM was maintained at a high level, and the average fluorescence intensity of EpCAM was significantly increased in all three kinds of sorted L‐CSCs (Fig. 3C–E). The expression proportion of CD133 in sorted Huh7.5.1 and Hep3B cells was also maintained at a high level, except that of PLC/PRF/5, but the average fluorescence intensity of CD133 was significantly decreased in all three kinds of sorted HCC cells (Fig. 3C–E).

In general, the modified CDM could stably maintain a high level of expression of CSC surface markers of enriched L‐CSCs, except CD133 expression of PLC/PRF/5.

### Compared with wild‐type HCC cells, the enriched L‐CSCs highly expressed stemness‐related oncogenes CK19, c‐MYC and SOX2

**Fig. 4:**
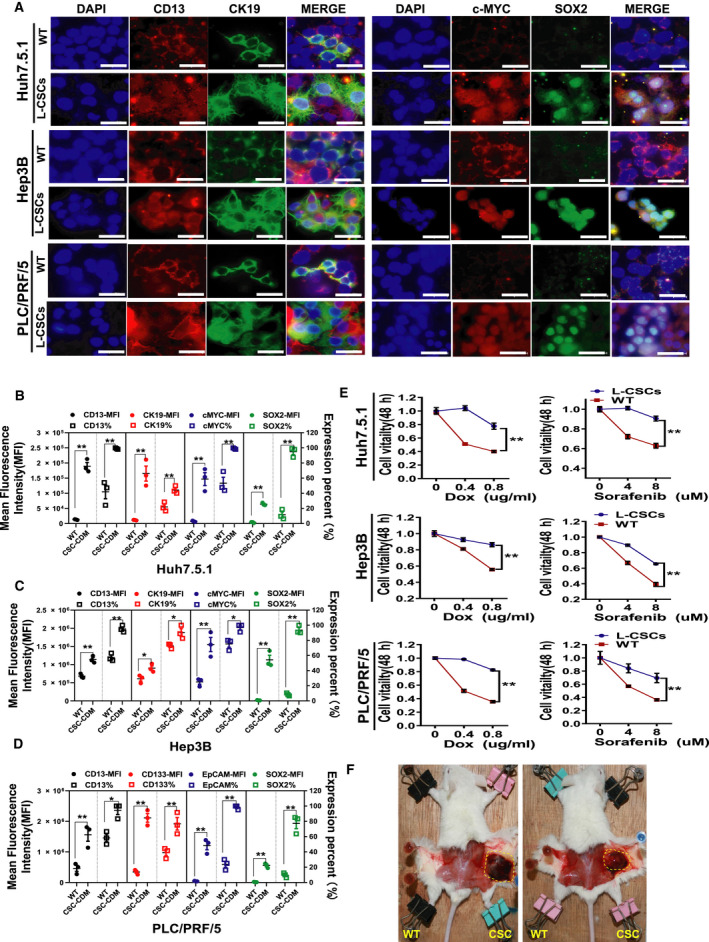
The enriched liver CSCs have stronger stemness‐related oncogenes expression, drug resistance and tumorigenicity than those of wild‐type HCC (WT). (A) Immunofluorescent images of CD13, CK19, c‐MYC and SOX2 in three enriched L‐CSCs (CSC‐CDM) or WT. Scale bars: 40 μm (B–D). The histograms of CD13, CK19, c‐MYC and SOX2 expression in three enriched L‐CSCs or wild‐type HCC cells. Mean ± SEM, *n* = 3. *P < 0.05; **P < 0.01, data analyzed by Student's *t*‐test. (E) The chemoresistance assay of doxorubicin (Dox) and sorafenib on enriched L‐CSCs or wild‐type HCC. Mean ± SEM, *n* = 3. *P < 0.05; **P < 0.01, data analyzed by two‐way ANOVA. (F) Gross observation of two mice with tumors derived from Hep3B‐CSCs cultured with CDM. Left flank, wild‐type Hep3B group; right flank, Hep3B‐CSC group; *n* = 3.

In addition to these liver CSC surface markers, we also detected other stemness gene expressions in enriched L‐CSCs, such as SOX2, c‐MYC and CK19. They were also oncogenes overexpressed in a variety of malignant tumors and play an important role in the function of L‐CSCs [17, 19, 20]. Immunofluorescence staining was used to detect these stemness‐related oncogene expressions of L‐CSCs and wild‐type HCC cells, and CD13^+^ HCC cells were used as a positive control group (Fig. 4A). The results showed that the expression proportion and MFI of CK19, c‐MYC and SOX2 in enriched L‐CSCs were significantly increased compared with those of wild‐type HCC cells (Fig. 4B–D).

### The enriched L‐CSC has a stronger drug resistance and tumorigenicity than the wild‐type HCC cells

Drug resistance is one of the characteristics of CSCs that has been reported by many researchers. Here, we compared the drug resistance of L‐CSCs cultured by CDM and wild‐type HCC cells. The cells were treated with different concentrations of Adriamycin or sorafenib for 48 h, and the cell viability of each group was detected by Cell Counting Kit‐8. The results showed that all three kinds of enriched L‐CSCs had a stronger drug resistance than their parental wild‐type HCC cells (Fig. 4E), which was in line with the characteristics of L‐CSCs. These results suggest that our model can be used for screening potential drugs targeting L‐CSCs *in vitro*.

Most important, we also compared the tumorigenicity of enriched L‐CSCs and wild‐type HCC cells *in vivo*. We injected 10 000 wild‐type Hep3B cells cultured in traditional serum‐contained medium and Hep3B‐CSCs cultured in CDM for 2 weeks into the left and right sides of the back of NOD/SCID mice, respectively (*n* = 3). Four weeks later, the results showed that there were two tumorigenic mice in the Hep3B‐CSC group, but no tumorigenic mouse in the wild‐type Hep3B group, indicating that the enriched L‐CSC has a stronger tumorigenicity than wild‐type HCC cells *in vivo*.

## Discussion

In this study, we used CD13, CD133 and EpCAM to separate and enrich a specific subpopulation of L‐CSCs and tried to use modified CDM to maintain their stemness‐related phenotypes. Although the results of different HCC cell lines have certain heterogeneity, the results show the same trend that the model can rather stably maintain CSCs phenotype of enriched L‐CSCs during the 2‐week culture process. Meanwhile, it does not significantly enhance the self‐renewal ability of non‐CSCs. Through the single‐cell *in situ* tracking assay, we have worked out the proportion of active Hep3B‐CSCs with long‐term clonal maintenance ability (about 1/8), which was 8.5 times higher than that of Hep3B‐non‐CSCs (1/68). Interestingly, the specific value was close to the ratio of spheres cross‐sectional area (C/D = 8.9) between Hep3B‐CSCs and Hep3B‐non‐CSCs in Fig. 2B. Through this method, we can compare the number of active CSCs with long‐term clonal maintenance ability in different subpopulations of CSCs *in vitro*, which is useful to analyze the relationship between different subpopulations of L‐CSCs and poor prognosis of patients.

There is still one issue of this model that needs to be proved. Our results suggested that culture CD13^+^CD133^+^EpCAM^+^ HCC cells with modified CDM could effectively maintain a high level of expression of CD13 and EpCAM in terms of both expression proportion and MFI. The CD133 expression proportion of enriched L‐CSCs was still higher than those of wild‐type HCC cells, but the MFI of CD133 was down‐regulated. The result may be because of a certain amount of BMP4 (10 ng·mL^−1^) in modified CDM. It has been reported that BMP4 could reduce the expression of CD133 in PLC/PRF/5‐CSCs with a dose‐ and time‐dependent manner [21]. Interestingly, they also found that low‐dose exogenous BMP4 could up‐regulate CD133 protein expression *in vitro*. Perhaps during the 2‐week culture process, the concentration of BMP4 in the enriched L‐CSCs is more than 10 ng·mL^−1^ due to the evaporation of the liquid and the regular replenishment of the CDM liquid, which results in the down‐regulation of CD133. In the next step, we will reduce the amount of BMP4 used in the culture process, especially in PLC/PRF/5 cells, which may have a better effect on the stable maintenance of these L‐CSC surface markers.

The significance of this work is to provide a rapid and reliable method to study a specific subpopulation of CSCs, not only limited to the CD13^+^CD133^+^EpCAM^+^ HCC, but also can be a combination of other CSC surface markers or other types of cancer. By adjusting different combinations of CSCs markers and the kinds or dosages of growth factors contained in CDM media, we hope to achieve *in vitro* culture and maintenance of different subpopulations of CSCs and provide an effective model for the study of CSC‐associated biological properties and CSC‐targeted drug screening.

## Conclusions

The enriched CD13^+^CD133^+^EpCAM^+^ HCC cells represented L‐CSC‐related functions and properties. After 14 days of modified CDM culture *in vitro*, the subpopulation of L‐CSCs still stably maintains CSC‐related phenotypes. This model provides a new method for L‐CSC research *in vitro* and the screening of CSC‐targeted drugs.

## Author contributions

WY and X‐TP supervised and coordinated all aspects of the work. BZ and H‐YW performed the experiments, analyzed data and prepared figures and tables. J‐NZ, D‐XW and ZF assisted with *in vivo* treatment and *in vitro* experiments. QZ helped with molecular experiments. J‐FX, XN and L‐JH provided technical or material support. BZ, J‐NZ and WY designed the research, analyzed data and wrote the manuscript.

## Conflict of interest

The authors declare no conflict of interest.

## Supporting information


**Fig. S1.** The single‐cell *in situ* tracking assay at 45‐ and 32‐cell gradients. (A, B) Long‐term clonal growth assay for single Hep3B‐CSC and Hep3B‐non‐CSC at 45‐ and 32‐cell gradients. Clonal islands 1–5 were formed by Hep3B‐CSCs at 45‐cell gradient (A). Clonal islands a–d were formed by Hep3B‐CSCs at 32‐cell gradient (B). Scale bars: 200 μM.
**Table S1.** The kits and reagents used in the study.
**Table S2.** The antibodies used in the study.

## Data Availability

The data and materials are available from the corresponding author on reasonable request.
