# Supplementary material for: A new protocol for long‐term culture of a specific subpopulation of liver cancer stem cells enriched by cell surface markers
Source: FEBS Open Bio. 2020 Jul 31;10(9):1737–47. doi: 10.1002/2211-5463.12932 (PMC7459405; doi:10.1002/2211-5463.12932)
Supplement: Supplementary file 1 — Fig. S1. The single‐cell in situ tracking assay at 45‐ and 32‐cell gradients. (A, B) Long‐term clonal growth assay for single Hep3B‐CSC and Hep3B‐non‐CSC at 45‐ and 32‐cell gradients. Clonal islands 1–5 were formed by Hep3B‐CSCs at 45‐cell gradient (A). Clonal islands a–d were formed by Hep3B‐CSCs at 32‐cell gradient (B). Scale bars: 200 μM. Table S1. The kits and reagents used in the study. Table S2. The antibodies used in the study. [file FEB4-10-1737-s001.docx]

**Supplementary Material**

**Supplementary Figure 1**


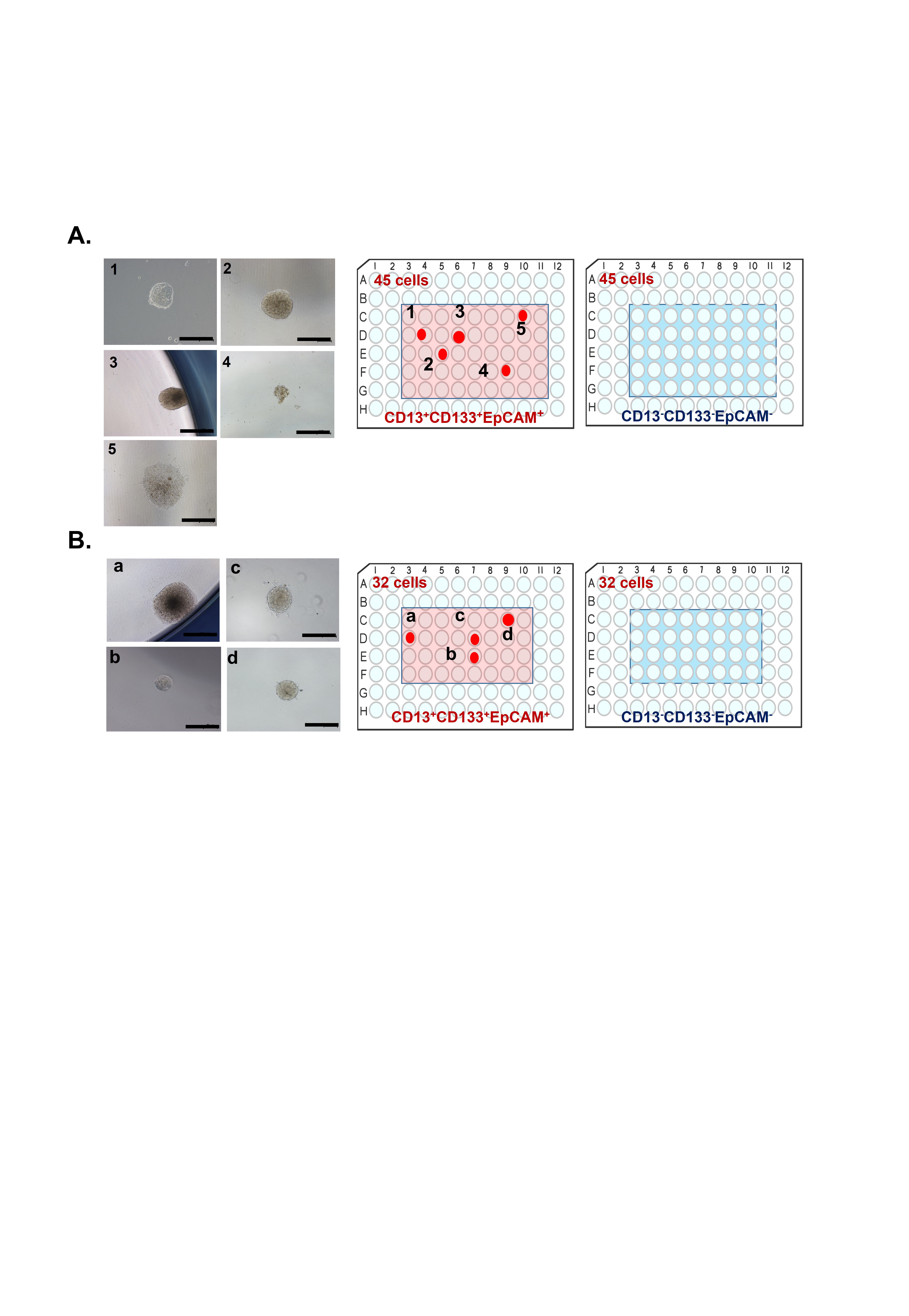


**Figure.S1 The single cell in situ tracking assay at 45 and 32 cells gradient.**

(A-B). Long-term clonal growth assay for single Hep3B-CSC and Hep3B-non-CSC cell at 45 and 32 cells gradient. Clonal islands 1-5 were fromed by Hep3B-CSCs at 45 cells gradient (A), Clonal islands a-d were fromed by Hep3B-CSCs at 32 cells gradient (B), Scale bars=200μM.

**Supplementary Table 1 The kits and reagents used in the study.**

| **Kits & Reagents** | **Applications** | **Catalog No.** | **Source** |
| --- | --- | --- | --- |
| FBS | cell culture | FSP500 | Excell Biology |
| DMEM-F12 | cell culture | 11330-057 | Life Technologies |
| DMEM | cell culture | D1152-1L | Sigma |
| MEM basic | cell culture | C11095500BT | GIBCO |
| Neurobasal Medium | cell culture | 21103-049 | GIBCO |
| Pen Strep | cell culture | 15140-122 | GIBCO |
| Bovine serum albumin | cell culture | V900933-100G | Sigma |
| N-2 | tumorshpere | 17502-048 | GIBCO |
| B-27 | tumorshpere | 17504-044 | GIBCO |
| L-Glutamine | cell culture | 11811-031 | GIBCO |
| rhHGF | cell culture | 294-HGN | R&D |
| rhBMP-4 | cell culture | 314-BP | R&D |
| EGF | tumorshpere | 236-EG-200 | R&D |
| b-FGF | tumorshpere | 100-18C | Peprotech |
| TGF-α | cell culture | 100-16A-100UG | Peprotech |
| Cell Counting Kit-8 | cell counting | CK04 | DOJINDO |
| Adriamycin (Dox) | drug resistance | A603456-0025 | Sangon Biotech |
| Sorafenib | drug resistance | sc-220125 | Santa Cruz Biotechnology |

**Supplementary Table 2 The antibodies used in the study.**

| **Antibody** | **Applications** | **Cat. No./Source** | **Origin** | **Dilution** | **Molecular weight/Localization** |
| --- | --- | --- | --- | --- | --- |
| PE-CD13 | FC | 555394, BD Pharmingen™ | mouse | 10μL /test | Cell membrane |
| PE-IgG Isotype | FC | 555574, BD Pharmingen™ | mouse | 10μL/test |  |
| PerCP-CyTM5.5-CD13 | FC | 561361, BD Pharmingen™ | mouse | 5μL/test | Cell membrane |
| PerCP-CyTM5.5-IgG Isotype | FC | 550795, BD Pharmingen™ | mouse | 5μL/test |  |
| PE-CD133 | FC | 130-080-801, Miltenyi Biotec | mouse | 5μL/test | Cell membrane |
| APC-EpCAM | FC | 324208, Biolegend | mouse | 5μL/test | Cell membrane |
| APC-IgG Isotype | FC | 400327, Biolegend | mouse | 5μL/test |  |
| CD13 | IF | ab7417, Abcam | mouse | 1:200 | Cell membrane/ cytoplasm |
| CK19 | IF | ab52625, Abcam | rabbit | 1:200 | 44 KD/Cytoplasm |
| Sox2 | IF | 3579S, CST | rabbit | 1:200 | 35 KD/ Nuclei |
| c-Myc | IF | 9E10, Santa | mouse | 1:400 | 57KD/Nuclei ,Cytoplasm |
